# Inversion of the Uterus

**Published:** 1864-02

**Authors:** 


*Inversion of the Uterus.*—The reduction of an inverted uterus has been considered always a difficult operation. The history and result, given in the interesting article of Dr. Eaton, in the present number, leads to a different conclusion. We congratulate the Doctor on the success of his enterprise and tact.

